# Calves peak-end memory of pain

**DOI:** 10.1038/s41598-023-32756-5

**Published:** 2023-04-07

**Authors:** Thomas Ede, Raphaela E. Woodroffe, Marina A. G. von Keyserlingk, Daniel M. Weary

**Affiliations:** 1 Animal Welfare Program, Faculty of Land and Food Systems, University of British Columbia, 2357 Main Mall, Vancouver, BC V6T 1Z6 Canada; 2 Present Address: Department of Clinical Studies, Swine Teaching and Research Center, School of Veterinary Medicine, University of Pennsylvania, Kennett Square, PA USA

**Keywords:** Neuroscience, Emotion

## Abstract

In humans, the ‘peak-end’ rule states that recollection of an experience is most often influenced by the peak (the most intense moment) and end of the experience. We investigated whether calves followed the peak-end rule in their memory of a painful procedure: disbudding. As proxies for retrospective and ‘real-time’ reports of pain, we used conditioned place aversion, and reflex pain behaviours. In two separate trials, calves were subjected to two disbudding conditioning sessions (one horn per treatment), acting as their own control. In the first trial, calves (n = 22) were disbudded and remained in a pen for 4 h, and disbudded and left in another pen for 4 h with an additional 2 h following an analgesic treatment. In the second trial, calves (n = 22) were disbudded and left in pens for 6 h during both treatments, receiving the analgesic at either 2 h or 4 h after disbudding. Calves were then tested for place aversion. For both trials we did not observe a preference for the pens where calves received analgesic treatment towards the end of the session. We did not find an association between aversion and the sum, peak or end of pain behaviours. Results are not consistent with a peak-end effect in calves’ memory of pain.

## Introduction

Retrospective reports are a crucial component of many fields of research. Yet memories of an experience often deviate from an accurate account of what is reported during the experience. Substantive research supports a ‘peak-end’ rule, noting that the peak (i.e. the most intense moment) and end of affective episodes are most influential in people’s recollection of the event^1,2^.

In a seminal study, Kahneman et al.^3^ subjected participants to two painful treatments: immersion of a hand in 14 °C water for 60 s, and the same immersion in 14 °C water for 60 s with an added 30 s in water raised to 15 °C. After being exposed to both treatments, participants were asked which one they would rather repeat. Surprisingly, most subjects chose to repeat the treatment including the additional time of milder pain; essentially ‘preferring more pain over less’. These results are inconsistent with a purely hedonistic view of behaviour, which would entail minimizing negative events and maximizing positive ones^2^. Linking participants’ retrospective evaluations with real-time discomfort ratings during treatments, Kahneman and colleagues proposed that this counterintuitive finding was due to participants giving more weight to the worst and final moments of painful experiences (‘peak-end’ rather than a sum of negative moments).

Since then, the peak-end rule has been observed in the evaluation of other painful episodes such as colonoscopies, lithotripsy^4^, arthritis^5^, headaches^6^ and childbirth^7^. Peak-end was also noted in the assessment of non-painful negative experiences such as annoying sounds^8^, poor air quality^9^ and a cognitively demanding task^10^. Peak-end appears to apply in a wide range of context, further reported in positive experiences such as receiving material goods^11^, food enjoyment^12,13^, browsing social media^14^, attending conference presentations^15^ and playing games^16^.

Although violations of hedonism are well studied in human pain research, no study has—to our knowledge—attempted to investigate the peak-end rule in pain processing of animals. The objective of this study was to assess whether dairy calves (*Bos taurus taurus*) follow the peak-end rule in their evaluation of painful experiences. The pain model selected was hot-iron disbudding, a routine procedure on dairy farms in which horn tissue is destroyed by cauterization^17^. Real-time and retrospective verbal patient reports usually used in human research are not available with non-human animals. As a proxy for real-time pain reports, we relied on calves' pain-related behaviours commonly used in the study of disbudding^18^. For retrospective reports, we adopted a place conditioning paradigm we previously used to assess the negative memory of hot-iron disbudding in dairy calves^19,20^.

The present study consisted of two trials, both subjecting calves to two disbudding treatments (one horn at a time). In both experiments, calves were given analgesics towards the end of one treatment, but not the other treatment. The first trial was designed to functionally mimic Kahneman et al.^3^: in one treatment, calves recovered from the procedure for 4 h without postoperative pain control. In the other treatment, calves went through the same 4 h recovery period, but this was followed by an additional 2 h after treatment with ketoprofen, a fast acting non-steroidal anti-inflammatory drug (NSAID)^21^ effective for mitigating this pain (Faulkner and Weary, 2000). To control for a potential effect of duration, a second trial was conducted in which both treatments had the same duration of 6 h. In one treatment, calves received the NSAID early (2 h post disbudding). In the other treatment, the NSAID was provided later (4 h post disbudding).

We predicted that (1) calves would display conditioned place aversion (i.e., a less favorable retrospective report) to the environment where they had not received analgesia towards the end of the experience, and (2) the highest and final numbers of pain behaviours (i.e., peak-end of real-time reports) would be better predictors of calves’ place conditioning responses than the sum of pain behaviours (hedonistic view).

If present in calves (and any species subjected to painful procedure), this cognitive bias could greatly influence how pain experienced by animals is considered in farming and veterinary practices. If maximum intensity and/or conclusive moments of painful experiences are the most impactful, pain control strategies that specifically target these periods would be most effective. Knowledge of the peak-end rule in farm animals could also facilitate stockmanship by improving animals’ recollection of routine, yet potentially negative events such as injections, artificial inseminations, hoof-trimming or first milking (and potentially many others).

## Results

### Place aversion

We found no evidence of difference in time spent in treatment pens during pre-exposure for either trial (Trial 1: t = 0.1, 95CI = [− 2.6, 2.9 min], *P* = 0.9; Trial 2: t = 0.1, 95CI = [− 1.5, 1.7 min], *P* = 0.9).

**Figure 1:**
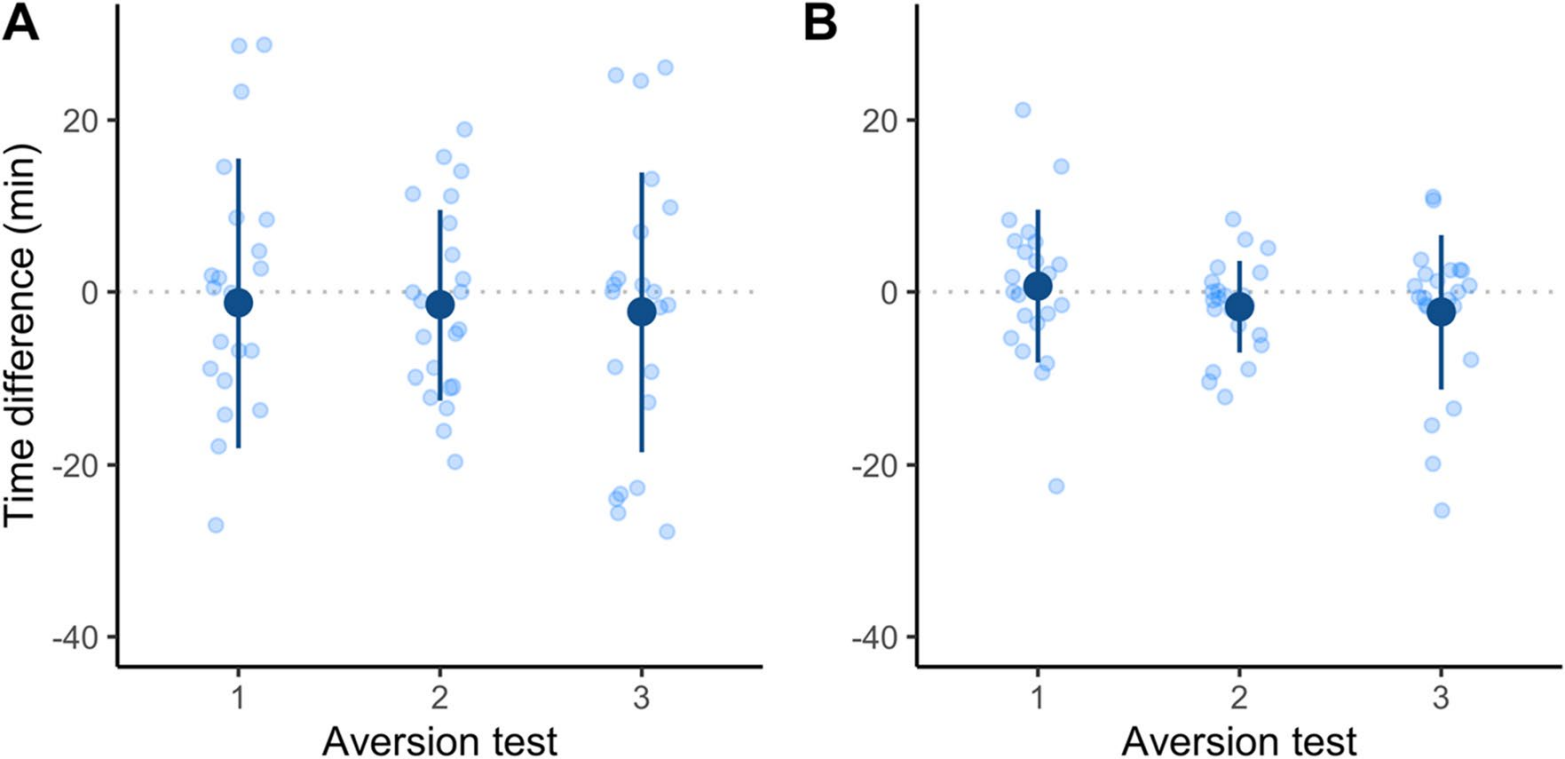
Conditioned place aversion results. Distribution of time differences spent in pens where calves (n = 22 for each trial) had previously been disbudded. Positive values reflect calves spending more time in a pen where they experienced analgesic effects towards the end of conditioning. In both trials, calves orally received the analgesic ketoprofen 2 h before exiting the apparatus. In trial 1 (**A**), it was compared to exiting the apparatus immediately after ketoprofen treatment. In trial 2 (**B**), it was compared to receiving ketoprofen 4 h before exiting the apparatus. Aversion tests 1, 2 and 3 took place 48 h, 72 h and 96 h after the last conditioning treatment. Dark blue circles and lines represent mean and standard error of the mean. Light blue circles represent individual values.

During aversion tests for Trial 1 (Fig. 1A), the intercept for difference in time spent between the two treatment pens was not significantly different from 0 (t = − 1.0, 95CI = [− 24.3, 7.1 min], *P* = 0.3). There was no effect of test session (t = − 0.3, 95CI = [− 4.4, 3.3 min], *P* = 0.8), treatment order (t = 1.5, 95CI = [− 1.5, 15.1 min], *P* = 0.1), pen colour (t = − 0.9, 95CI = [− 12.2, 4.3 min], *P* = 0.4) or horn side (t = − 0.1, [− 8.7, 7.9 min], *P* = 0.9). Excluding all non-significant fixed effects, no difference was found in time spent between pens during aversion tests (t = − 0.8, 95CI = [− 6.1, 2.8 min], *P* = 0.5). There was also no difference in the location calves chose to lie down in relation to the treatment they had previously received (X^2^ = 0.1, *P* = 0.8).

During aversion tests for trial 2 (Fig. 1B), we also did not find a difference in time spent in the two treatment pens (t = 0.5, 95CI = [− 7.0, 11.4 min], *P* = 0.7). There was no effect of test session (t = -1.4, 95CI = [− 3.5, 0.5 min], *P* = 0.1), treatment order (t = − 0.1, 95CI = [− 4.4, 5.0 min], *P* = 0.9), pen colour (t = − 0.9, 95CI = [− 7.0, 2.4 min], *P* = 0.4) or horn side (t = 0.2, [− 4.1, 5.2 min], *P* = 0.8). Excluding all non-significant fixed effects, no difference was found in time spent between pens during aversion tests (t = − 0.9, 95CI = [− 3.5, 1.3 min], *P* = 0.4). There was no difference in the location calves chose to lie down in relation to the treatment they had received (X^2^ = 0.2, *P* = 0.7).

### Predictive values of pain behaviours

**Figure 2:**
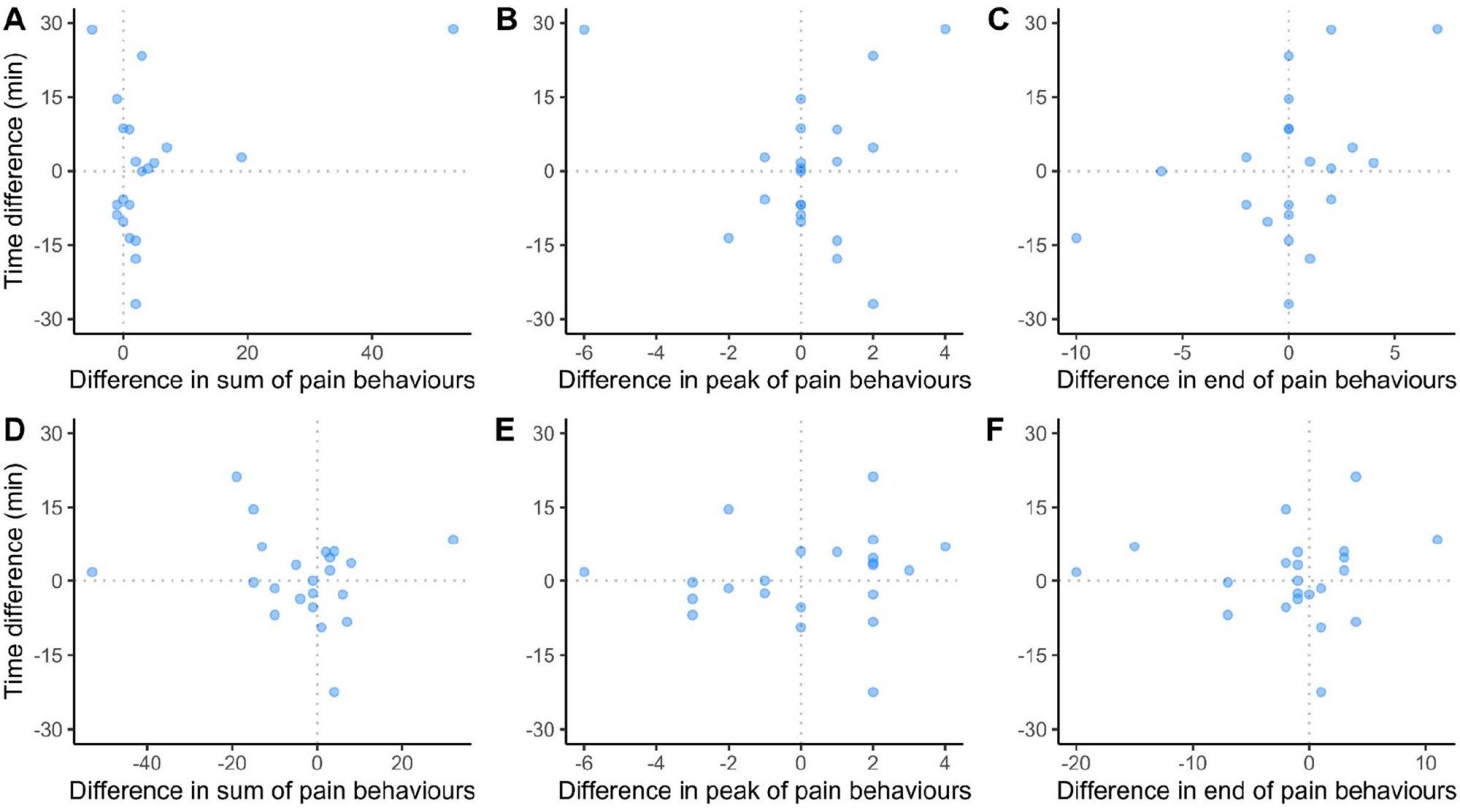
Correlations between aversion results and pain behaviours for Trial 1 (**A**–**C**) and Trial 2 (**D**–**F**). Correlations were tested for the ‘sum’ (pain behaviours displayed over the whole treatment duration, **A**, **D**), ‘peak’ (highest occurrence, **B**, **E**) and ‘end’ (over the last two hours, **C**, **F**).

We found no evidence of an association between aversion and ‘sum’, ‘peak’ or ‘end’ pain behaviours (Fig. 2), in either Trial 1 (sum: t = 1.9, 95CI = [− 0.04, 0.7], *P* = 0.07; peak: t = 0.2, 95CI = [− 0.4, 0.5], *P* = 0.8; end: t = 1.7, 95CI = [− 0.09, 0.7], *P* = 0.1), or Trial 2 (sum: t = − 0.8, 95CI = [− 0.5, 0.3], *P* = 0.5; peak: t = 0.5, 95CI = [− 0.3, 0.5], *P* = 0.6; end: t = 0.2, 95CI = [− 0.4, 0.5], *P* = 0.9).

## Discussion

Calf aversion was not influenced by timing of analgesia. Contrary to our predictions, we did not observe a preference for the pen where calves had received analgesic treatment towards the end of the experience. This was the case when time was added at the end (Trial 1) and when the duration was balanced between treatments (Trial 2). Moreover, pain behaviours (sum, peak or end) were not associated with aversion.

Our results are not consistent with the strong support for the peak-end rule in humans^1^. That said, some previous research in humans has also found that peak-end effects are sometimes poor predictors of past affective experiences. For example, students did not follow the peak-end rule in their recall of vacations^22^, consumers evaluations did not show a superiority of peak-end in their evaluation of tourism advertisements^23^, no peak-end effect was observed when children or adults received small gifts such as candies and toys^24^, and people’s evaluation of a previous day relied on average ratings rather than peaks or ends^25^. Although seldom explored in animals, peak-end was not observed in a small study of 3 rhesus macaques^26^.

There were several limitations to the current study that may have affected the results. The paradigm used in this study was successful in comparing calves’ aversion responses to procedures with a strong contrast: disbudding vs a sham procedure^19^ and disbudding with or without post-operative analgesia^20^, but our method for assessing aversion might not be sufficiently sensitive to assess more subtle differences^27^.

As calves received milk rewards during conditioning sessions, these positive events might have reduced the negative association between pen and painful experience. Conversely, it is also possible that hunger levels negatively influenced how calves perceived their experience, although no aversion was found to the pen where calves remained two additional hours (Trial 1, Treatment ‘6 h’, see Fig. 4). The long conditioning sessions (from 4 to 6 h) might have made the calves too accustomed to the pens to establish a specific association with their pain experience.

As we did not find a correlation between the sum of pain behaviours and place conditioning results, our study also does not support an additive hedonic account of pain memory. It is unclear whether this lack of difference is due to untested cognitive processes from calves or methodological issues of sensitivity from aversion test and/or pain behaviours as measures for affective states.

Although the pain behaviours used in this study (ear flicks, head rubs and head shakes) have previously been reported as good indicators of ketoprofen treatment following disbudding^28^, other authors did not observe an effect of ketoprofen on pain behaviours in the 8 h after disbudding^29^. In a recent meta-analysis of the effects of local anesthesia and NSAID on disbudding, behavioural responses were found to be heterogeneous^18^. More generally, spontaneous behavioural responses might be weak predictors of affective states. Concerns over the value of reflex responses have previously been raised, noting the presence of such responses in the absence of felt emotions^30^, or that pain behaviours could reflect minor irritation to extreme pain^31^.

Previous work done in our laboratory has shown that oral administration of ketoprofen can be successful^28^, and this delivery method can avoid any effect of painful injections^32^ on the calves’ memory of the event. However, the pharmacokinetics of ketoprofen administered orally remain largely unknown. Most research has focused on intramuscular (IM) and intravenous (IV) routes, finding ketoprofen to be effective in reducing mechanical nociceptive threshold in 1 h and 2 h post-dehorning (IM, 3 mg/kg)^33^ and having an elimination half-life in calves between 1 and 2 h (IV, 0.5–1.5 mg/kg)^34,35^ sometimes even shorter than 1 h (IV, 3 mg/kg)^21^. We assumed ketoprofen would have a similarly quick onset and metabolization when administered orally, but this assumption is untested.

In summary, we attempted to study the peak-end rule in dairy calves with conditioned place aversion and reflex behaviours as proxy measures of retrospective and ‘real-time’ reports of pain. We found no evidence for a peak-end effect in calves’ memory of pain. This result suggests a species difference between how cattle and humans remember painful events, and the need to refine methods of affect assessment in animals.

## Methods

### Animals

Studies were conducted from September 2020 to February 2021 at The University of British Columbia’s Dairy Education and Research Center in Agassiz, Canada. All procedures were approved by the university’s Animal Care Committee (Application A16-0310) and conducted in accordance with guidelines form the Canadian Council of Animal Care^36^. Reporting followed ARRIVE guidelines (see supplementary material for details).

A power analysis using R’s power.t.test function^37^ based on Kahneman’s^3^ study and results from a previous place conditioning study on ketoprofen^20^ was conducted. From these calculations, a minimum sample size of 18 per trial was determined.

A total of 44 Holstein calves (30.5 ± 6.5 d old) were enrolled in two separate trials (22 calves in each). All calves were housed individually from birth and moved to a group pen at 5 d old, where they were then housed in groups of 8 to 10. Calves had a daily milk allowance of 12 L, provided through an automatic feeder (CF 1000 CS Combi; DeLaval Inc., Sweden). Calves had ad libitum access to water, hay and grain through automatic bins and dispenser (RIC; Insentec B.V., Netherlands).

### Apparatus

**Figure 3:**
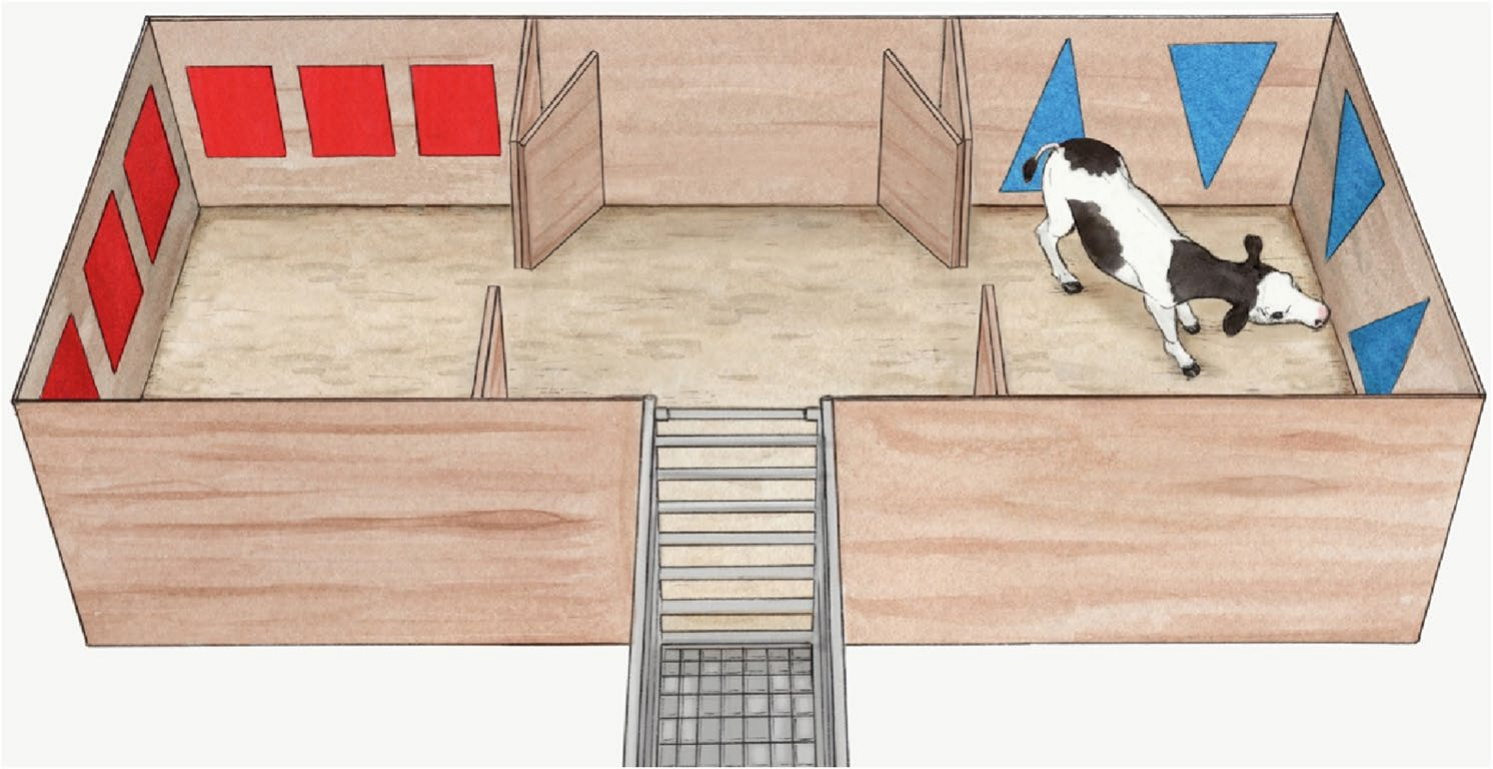
Conditioned place aversion apparatus. Each calf received and recovered from two different disbudding procedures in the treatment pens (side pens with visual cues on walls). After receiving both treatments, they were tested for place aversion by letting them roam between the pens. Illustration by Ann Sanderson.

**Figure 4:**
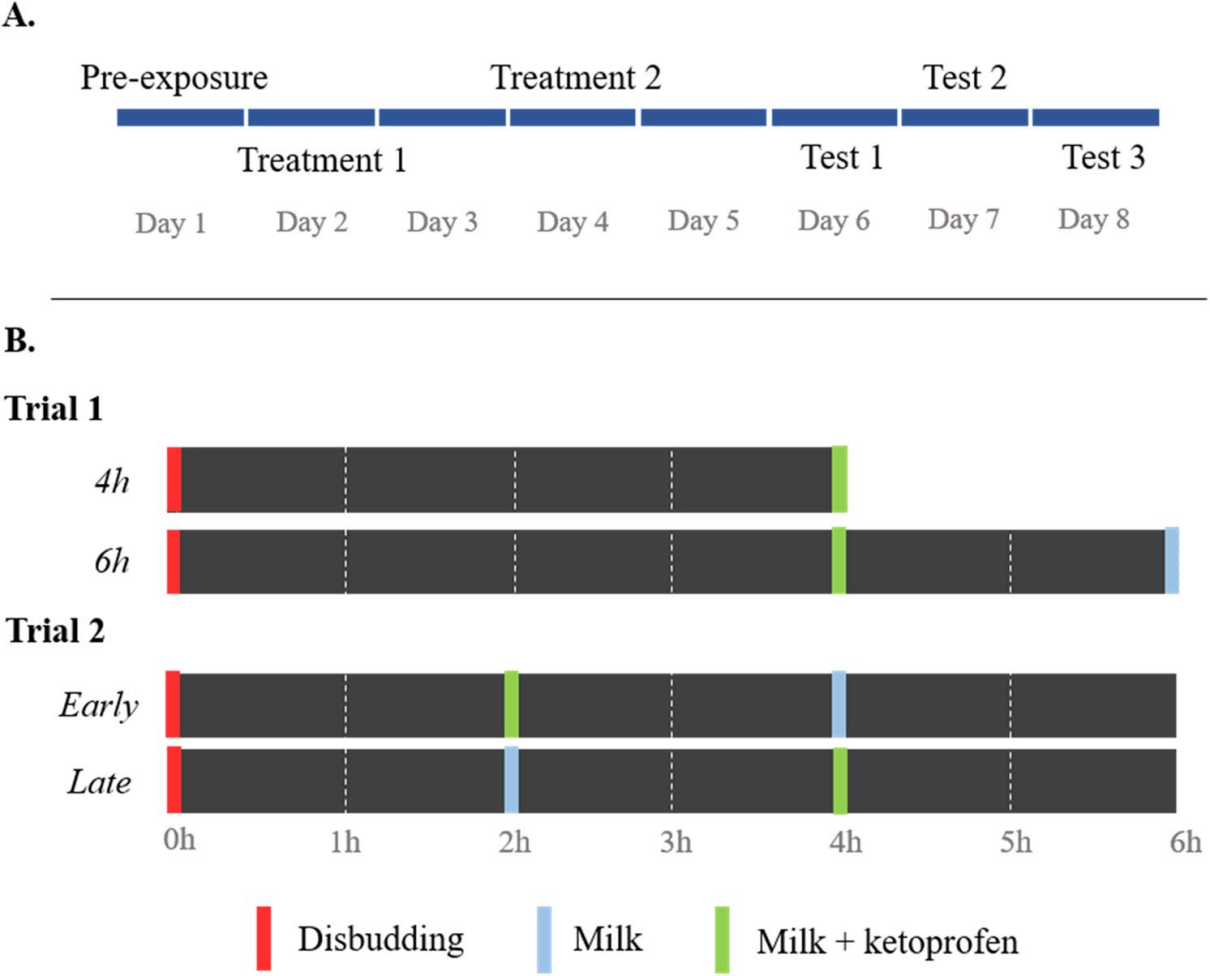
For both trials, calves received two conditioning sessions (‘Treatment 1 and 2’, Fig. 2A): one where an analgesic (ketoprofen) was provided towards the end of the experience, and one without. Calves were then tested three days in a row for place aversion. For trial 1, calves were left for an additional 2 h in the conditioning pen following ketoprofen treatment. For trial 2, calves received ketoprofen 4 h after disbudding instead of 2 h.

The place aversion apparatus was a 2.1 × 6.0 m pen divided in 3 sawdust-bedded areas: the middle pen, and two treatment pens (Fig. 3). Walls of treatment pens were fitted with coloured sheets cut into shapes: red squares on one side, blue triangles on the other. These visual cues were intended to help calves make the association between pen and treatment. A chute where calves were brought to at the beginning of each session was positioned in front of a gate leading into the middle pen. Gates separating the middle pen from treatment pens were removable. All experimental phases (pre-exposure, treatments and tests, see Fig. 4A) were recorded using cameras (WV-CP310, Panasonic Canada, Ontario) mounted over the apparatus. The apparatus was located indoors, in a closed and secluded room which allowed for constant ambient conditions.

### Pre-exposure

Calves had to fulfill a pre-exposure criterion to be enrolled (similar for trials 1 and 2). Calves were brought to the apparatus (which had the gates removed), and left to explore for 15 min. To avoid including calves with a strong pre-existing side bias, calves (n = 6) who did not enter both treatment pens during this time were not enrolled in the study. Another 4 fell ill during the study and were not included. A total of calves 44 calves were successfully enrolled (22 for each trial).

### Treatments

For both trials, calves acted as their own control by receiving two disbudding treatments: one per horn bud and in each treatment pen. Treatments were pseudo-randomly allocated, balancing pen, order, horn side, and pre-exposure preference using a Latin-square design. Calves received their first treatment 24 h after pre-exposure; and their second treatment 48 h after first treatment (allowing for a day of rest between treatments).

During treatment days milk delivery was suspended at approximately 9 AM. One hour later, calves were brought to the apparatus’ chute (Fig. 3) and offered 0.5 L of milk in a bottle. While in the chute, calves were injected with a sedative (xylazine 0.2 mg/kg, Rompun 20 mg/mL, Bayer, Leverkusen, Germany) and then led into the assigned treatment pen. As xylazine sedative effects last approximately 1 h in calves^38^, they were not expected to be affected at the time of aversion testing. Once the calf showed evidence of sedation (i.e., recumbent with slight eyeball rotation; approximately 10 min after xylazine injection), a cornual nerve block was administered (5 mL, lidocaine 2%, epinephrine 1:100,000, Lido-2, Rafter8, Calgary, AB, Canada). Ten min after the lidocaine injection the horn bud was shaved and tested for absence of pain reflex with a needle prick. The horn bud was then cauterized using a preheated hot-iron (X30, 1.3 cm tip, Rhinehart, Spencerville, IN), applying the tip for 10–20 s around the horn bud. Calves were then left in the treatment pen for the next 4 h or 6 h and were bottle-fed 0.5 L milk reward (with or without an analgesic) by experimenters via a rubber nipple at intervals depending upon treatment (see next section). Calves were then brought back in their home pen and allowed full access to their normal daily milk ration via the automatic milk feeder. Treatments (disbuddings and milk feedings) were conducted by TE and RW.

- Trial 1

During both treatments of Trial 1, calves received a 0.5 L milk reward with an analgesic mixed in (ketoprofen, 3 mg/kg, Anafen, 100 mg/mL, Boehringer Ingelheim, Ontario, Canada) 4 h after disbudding. During the ‘4 h’ treatment, calves were then immediately brought back in their home pen. During the ‘6 h’ treatment, calves were left an additional 2 h in the treatment pen, before receiving a 0.5 L milk reward (so both treatments would end with a milk reward) and brought back in their home pen (Fig. 4B).

- Trial 2

During both treatments of Trial 2, calves were left in the pen for 6 h after disbudding and received 0.5 L milk rewards at 2 h and 4 h after the procedure. During the ‘Early’ treatment, an analgesic (ketoprofen, 3 mg/kg, Anafen, 100 mg/mL, Boehringer Ingelheim, Ontario, Canada) was mixed in the milk offered 2 h after disbudding. During the ‘Late’ treatment, the analgesic was mixed in the offered 4 h after disbudding (Fig. 4B).

### Tests

Two days after the second treatment, calves were tested for conditioned place aversion. Aversion tests were similar to pre-exposure sessions in that calves were brought to the apparatus and could roam freely between the three pens (middle and both treatment pens). Test sessions lasted for a maximum of 60 min, or until the calf lay down for at least 1 min. If a calf did not lie down after 60 min in the apparatus, her latency to lay down was recorded as 1 h. The calf was then brought back to her home pen. Tests were conducted three days in row (Fig. 4A).

### Pain behaviours

Pain-related behaviours in the treatment pens following disbudding were video analysed using 1 min scan sampling every 10 min. A total of 2992 observations were split between 5 blinded observers (JK, SK, ZO, YR and LS). Behaviours were selected and defined based on previous research on disbudding pain^39^, see Table 1 for details. Pain behaviours were summed by observation, and reliability between observers was tested on a sample of 100 randomly selected observations. Using R’s ‘ICC’ function, inter observer reliability was rated as good (Intraclass correlation coefficient ≥ 0.8)^40^.

**Table 1:** Description of pain behaviours observed following hot-iron disbudding. Based on Duffield et al. (2010).

Behaviour	Description
Ear flick	Calf rapidly moves 1 or both ears to the front and back, independent of a head shake
Head rub	Calf scrapes her head against a wall of the pen or with a hind leg
Head shake	Calf rapidly shakes her head from side to side

### Statistical analysis

For both trials, differences in time spent in the treatment pens during aversion tests were analysed using linear mixed models^41^. Models included as fixed factors: aversion test session (1, 2 or 3, 1 df), treatment order (1 df), treatment pen colour (1 df) and horn side (1 df). Calf was included as a random factor (n = 22 for each trial). Significance of factors were calculated through p-values and 95% confidence intervals using R’s *lmerTest* package and base *confint* function^37,42^. Additional simplified models which did not include non-significant fixed effects were conducted. For all models, normality and homoscedasticity of residuals were confirmed graphically. Which pen calves chose to lay down in was analysed with Χ^2^ tests.

Due to the low number of individual measures, pain behaviours (ear flicks, head rubs and head shakes) were summed to form a single ‘pain behaviour’ measure. For each trial and calf, pain behaviours observed in two pens were summed over the entire observation period (i.e. the overall hedonic value), the highest frequency observed (i.e. the peak) and the last two hours (i.e. the end). Pearson’s correlation was used to assess the relation between aversion and the sum, peak and end behaviours. We limited correlations to the first aversion test session to avoid any potential influence of extinction^19^.

## Supplementary Information

Supplementary Information 1.

Supplementary Information 2.

Supplementary Information 3.

## Data Availability

Data and R code are freely accessible in supplementary materials.
