# Supplementary material for: Calves peak-end memory of pain
Source: Sci Rep. 2023 Apr 7;13:5679. doi: 10.1038/s41598-023-32756-5 (PMC10082038; doi:10.1038/s41598-023-32756-5)

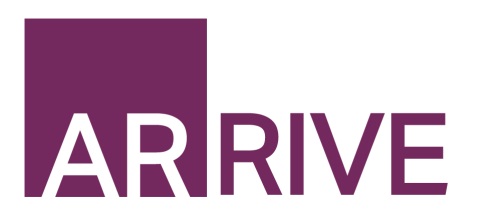


The ARRIVE Guidelines Checklist

Animal Research: Reporting In Vivo Experiments

Carol Kilkenny^1^, William J Browne^2^, Innes C Cuthill^3^, Michael Emerson^4^ and Douglas G Altman^5^

*^1^The National Centre for the Replacement, Refinement and Reduction of Animals in Research, London, UK, ^2^School of Veterinary Science, University of Bristol, Bristol, UK, ^3^School of Biological Sciences, University of Bristol, Bristol, UK, ^4^National Heart and Lung Institute, Imperial College London, UK, ^5^Centre for Statistics in Medicine, University of Oxford, Oxford, UK.*

|  | | ITEM | RECOMMENDATION | Page, § |
| --- | --- | --- | --- | --- |
| 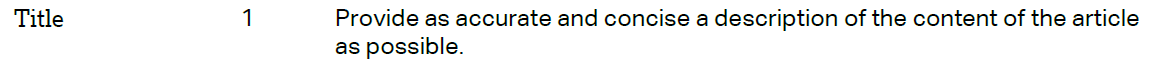 | | | p.1 |  |
| 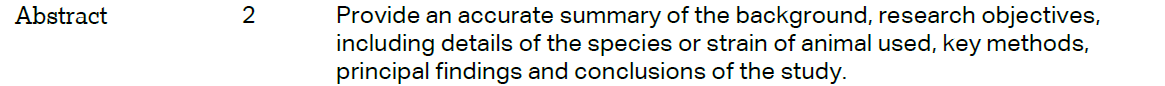 | | | p.2 |  |
| INTRODUCTION | | |  |  |
| 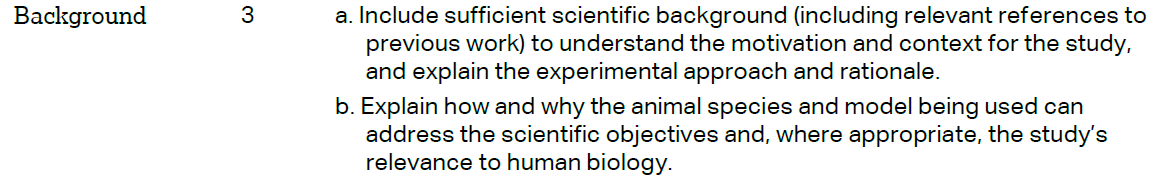 | | | p.3  p.3-4 |  |
| 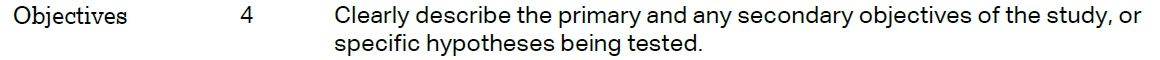 | | | p.3-4 |  |
| METHODS | | |  |  |
| 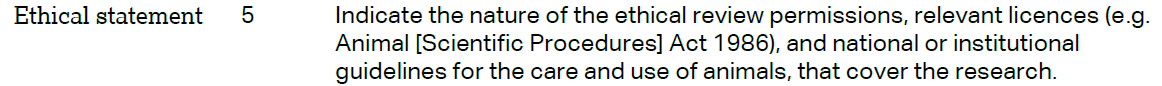 | | | p.8 |  |
| 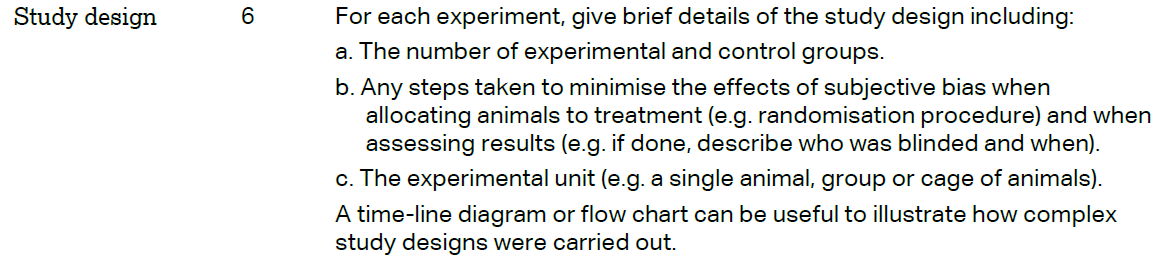 | | | p.8  p.9, 12  p.9,12, fig.4 |  |
| 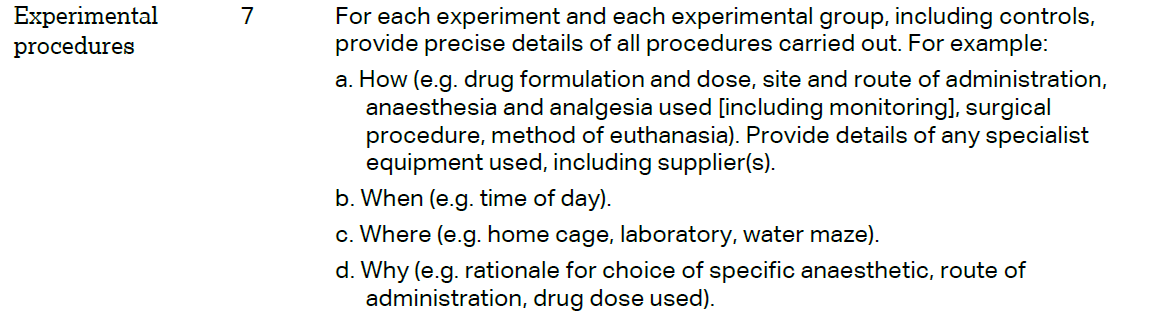 | | | p. 9-12  p. 10,11  p. 8-11  p. 4,7,8,10 |  |
| 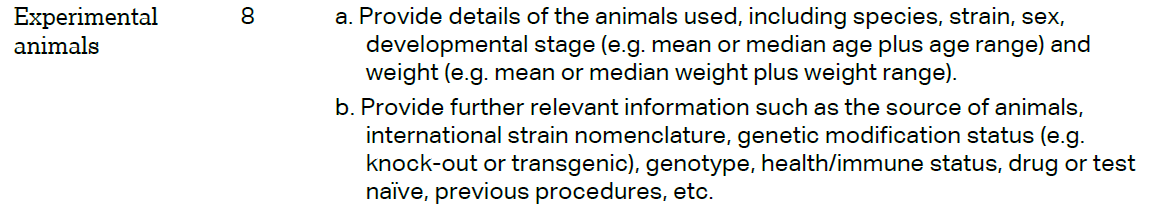 | | | p.8  p.8 |  |

The ARRIVE guidelines. Originally published in *PLoS Biology*, June 2010^1^

| 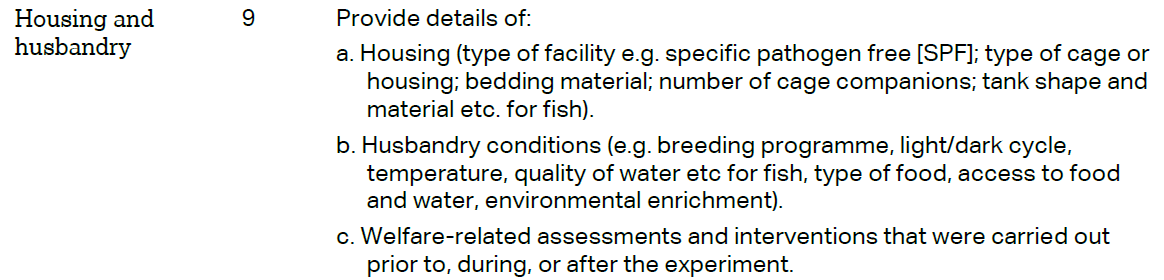 | p.8    p. 8  p.9,10,12 | |
| --- | --- | --- |
| 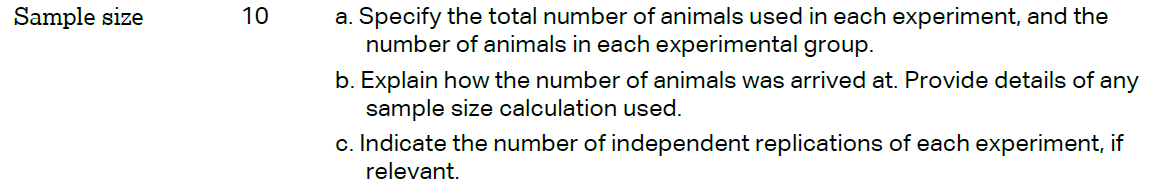 | p.8,9,12  p.8  NA | |
| 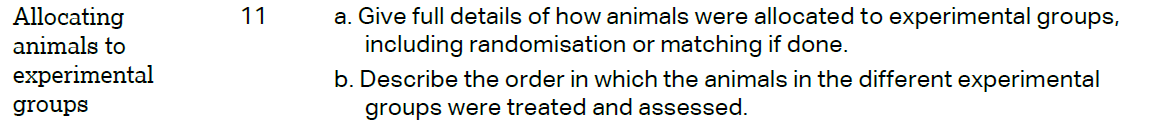 | p.9  NA | |
| 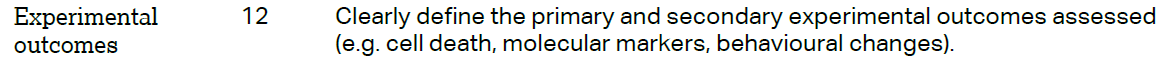 | p.4,11,12 | |
| 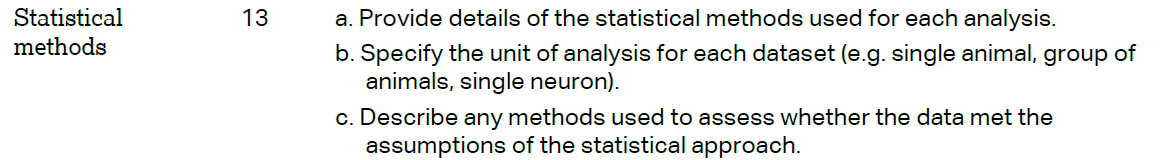 | p.12,13  p.12  p.12 | |
| RESULTS |  | |
| 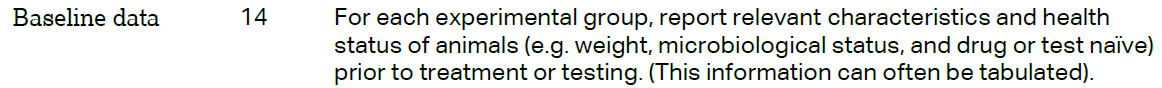 | p.4,8 | |
| 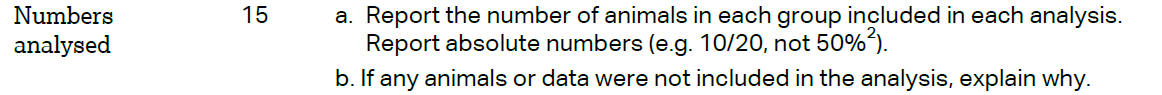 | p.8.9,12 | |
| 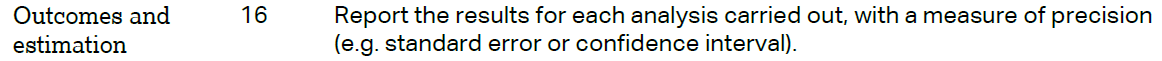 | p.4,5,6, fig.1,2 | |
| 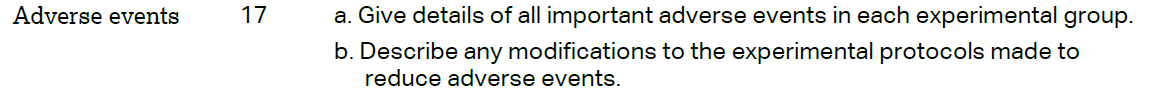 | p.9-11 | |
| DISCUSSION |  | |
| 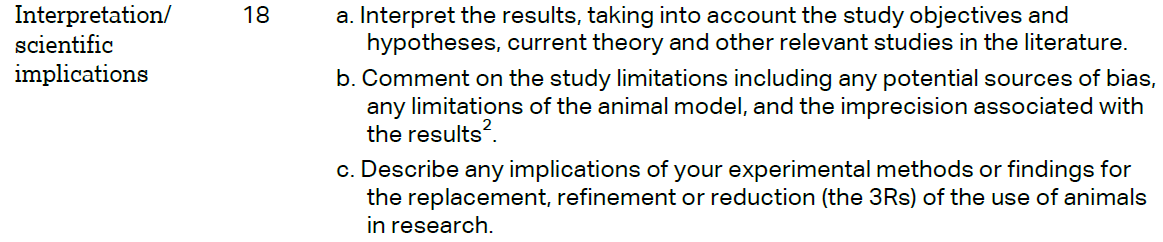 | p.6-8  p.7,8  p.7,8 | |
| 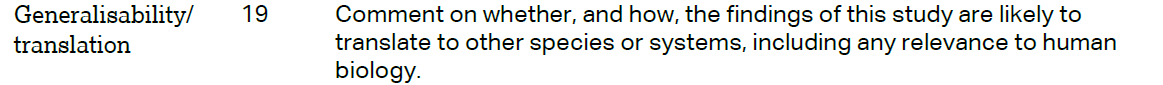 | NA | |
| 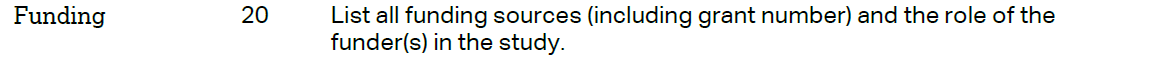 | | p.16,17 |


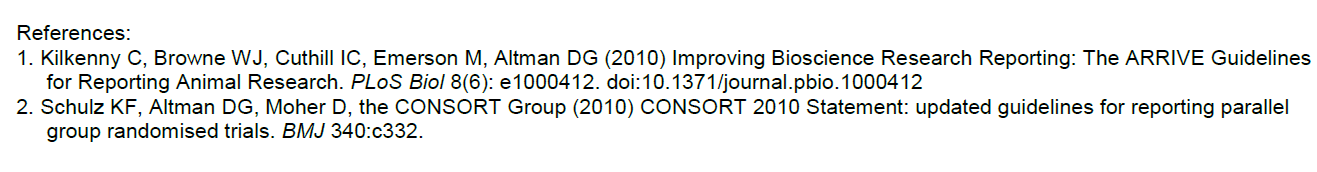

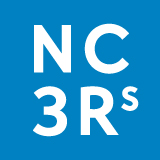

Supplement: Supplementary file 1 — Supplementary Information 1. [file 41598_2023_32756_MOESM1_ESM.docx]
