# Supplementary material for: Calves peak-end memory of pain
Source: Sci Rep. 2023 Apr 7;13:5679. doi: 10.1038/s41598-023-32756-5 (PMC10082038; doi:10.1038/s41598-023-32756-5)
Supplement: Supplementary file 3 — Supplementary Information 3. [file 41598_2023_32756_MOESM3_ESM.docx]

#R code Calves peak-end memory of pain - 2022 Scientific Reports

rm(list = ls())

library(ggplot2)

library(lme4)

library(lmerTest)

data = read.csv("/Users/thomasede/Desktop/Research/Peak end pain/Article/SciReports/data.csv")

attach(data)

#4h/6h----

data_4h6h = subset(data, Trial == "4h_6h")

attach(data_4h6h)

ggplot(data_4h6h, aes(x = factor(Session_n), y = tdiff/60))+

geom_boxplot()

lm = lmer(tdiff/60~Session_n+Order_6horLate+Pen_6horLate+Horn_6horLate+(1|factor(ID)))

plot(lm)

summary(lm)

confint(lm)

#Early/Late

data_Early_Late = subset(data, Trial == "Early_Late")

attach(data_Early_Late)

ggplot(data_Early_Late, aes(x = factor(Session_n), y = tdiff/60))+

geom_boxplot()

lm = lmer(tdiff/60~Session_n+Order_6horLate+Pen_6horLate+Horn_6horLate+(1|factor(ID)))

plot(lm)

summary(lm)

confint(lm)

#Correlations with pain behaviours----

attach(data_4h6h)

plot46sum = ggplot(data_4h6h, aes(x = sum_pbdiff, y = tdiff))+

geom_point()

plot46peak = ggplot(data_4h6h, aes(x = peak_pbdiff, y = tdiff))+

geom_point()

plot46end = ggplot(data_4h6h, aes(x = end_pbdiff, y = tdiff))+

geom_point()

attach(data_Early_Late)

plotELsum = ggplot(data_Early_Late, aes(x = sum_pbdiff, y = tdiff))+

geom_point()

plotELpeak = ggplot(data_Early_Late, aes(x = peak_pbdiff, y = tdiff))+

geom_point()

plotELend = ggplot(data_Early_Late, aes(x = end_pbdiff, y = tdiff))+

geom_point()

ggarrange(plot46sum,plot46peak,plot46end,plotELsum,plotELpeak,plotELend)

attach(data_4h6h)

cor.test(tdiff, sum_pbdiff)

cor.test(tdiff, peak_pbdiff)

cor.test(tdiff, end_pbdiff)

attach(data_Early_Late)

cor.test(tdiff, sum_pbdiff)

cor.test(tdiff, peak_pbdiff)

cor.test(tdiff, end_pbdiff)
